# Parathyroid Hormone in Pregnancy: Vitamin D and Other Determinants

**DOI:** 10.3390/nu13020360

**Published:** 2021-01-25

**Authors:** Ola Hysaj, Patricia Marqués-Gallego, Aline Richard, Magdeldin Elgizouli, Alexandra Nieters, Katharina C. Quack Lötscher, Sabine Rohrmann

**Affiliations:** 1 Epidemiology, Biostatistics and Prevention Institute, University of Zurich, Hirschengraben 84, 8001 Zurich, Switzerland; ola.hysaj@uzh.ch (O.H.); p.marquesgallego@gmail.com (P.M.-G.); aline.richard@uzh.ch (A.R.); 2 FREEZE-Biobank, Center for Chronic Immunodeficiency, Medical Center, Faculty of Medicine, University of Freiburg, Breisacherstr. 115 4, D-79106 Freiburg, Germany; elgizouli@medgen.uzh.ch (M.E.); alexandra.nieters@uniklinik-freiburg.de (A.N.); 3 Clinic of Obstetrics, University Hospital Zurich, Frauenklinikstrasse 10, 8091 Zurich, Switzerland; Katharina.QuackLoetscher@usz.ch

**Keywords:** parathyroid hormone, vitamin D, pregnancy, determinants

## Abstract

We aimed to assess the parathyroid hormone (PTH) concentration in pregnant women at the beginning of pregnancy (1st trimester) and within days before delivery (3rd trimester) and evaluate its determinants. From September 2014 through December 2015 in a cross-sectional study, 204 women in the 1st trimester of pregnancy and 203 women in the 3rd trimester of pregnancy were recruited. Blood samples were collected to measure PTH and circulating 25-hydroxy-vitamin D (25(OH)D) concentrations. Lifestyle and demographic data were collected using a questionnaire. Serum 25(OH)D and PTH were inversely correlated in both early and late pregnancy. Our analyses suggest that in the 3rd trimester of pregnancy, a 25(OH)D level of 18.9 ng/mL (47.3 nmol/L) could serve as an inflection point for the maximal suppression of PTH. Statistically significant determinants of PTH concentrations in multiple regression were 25(OH)D concentrations, season, multiparity and education of the partner (all *p* < 0.05) in early pregnancy. In late pregnancy, 25(OH)D concentrations and country of origin were statistically significant determinants of PTH concentrations (all *p* < 0.05). These factors and their effect on PTH appear to be vastly determined by 25(OH)D; however, they might also affect PTH through other mechanisms besides 25(OH)D.

## 1. Introduction

Physiological changes during gestation aimed at providing sufficient calcium for the growth of the fetus have been investigated extensively due to the health implications for both mother and offspring [1,2,3,4,5,6]. Calcium homeostatic hormones, such as parathyroid hormone (PTH), are essential to increase maternal calcium absorption during pregnancy [1]. PTH is involved in several processes, in particular maintenance of ionized calcium in the blood, raising the level of calcium phosphate released from bone tissue, conserving calcium, reducing tubular phosphate reabsorption, and increasing the intestinal absorption of calcium through vitamin D. Generally, in the first trimester of pregnancy, PTH concentrations are suppressed into the low normal range and may even decline below the normal range [7,8]. Through the course of pregnancy, PTH concentrations increase to reach a mid-normal range by the third trimester [1]. The secretion of PTH is regulated primarily by extracellular calcium concentrations; lower amounts of circulating calcium trigger an increase in PTH [1]. A rise in PTH-related protein (PTHrP) and calcitriol in the first trimester, and the flux of other hormones during pregnancy, such as estradiol, progestins, placental lactogen, and insulin-like growth factor I, may have direct or indirect effects on maternal calcium [9]. These changes might account for the observed differences in PTH concentrations in the first and third trimesters. Additionally, nephrogenous cyclic adenosine monophosphate (cAMP) excretion, the index of parathyroid function, has been reported to decrease during the first and second trimesters of pregnancy but to be in normal range during the third trimester [10].

The transfer of calcium to the fetus increases in the case of maternal hyperparathyroidism and hypercalcemia, with consequent suppression of fetal parathyroid glands [11]. Maternal hyperparathyroidism and hypercalcemia are known to result in a high rate of fetal complications: spontaneous abortions, intrauterine growth retardation, still births, transient neonatal tetany, and permanent hypoparathyroidism in the newborn [12,13]. Conversely, the placental transfer of calcium decreases in the case of maternal hypoparathyroidism and hypocalcemia, with the consequent stimulation of fetal parathyroid glands and the development of fetal hyperparathyroidism. Severe cases of fetal hyperparathyroidism suffer subperiosteal bone resorption, bowing of the long bones, intrauterine rib and limb fractures, low birth weight, osteitis fibrosa cystica, spontaneous abortion, and possibly fetal death [14,15,16,17]. Thus, it is highly essential to understand the determinants of adequate PTH concentrations during pregnancy. The effects of dietary and plasma calcium and plasma 25-hydroxyvitamin D (25(OH)D) on PTH have been well studied [18,19]. Although other determinants that might influence PTH levels have been suggested by prior research in the general population, such as the body mass index (BMI), ethnicity, smoking, season, and age, research on PTH determinants in pregnant women is scanty [2,3,18,20,21,22,23,24,25,26,27,28,29]. Correlation between PTH and 25(OH)D levels has been the main focus of studies in women of childbearing age and less frequently during pregnancy [21,23,24,25,26,27].

Given the implications of PTH on maternal and neonatal outcomes and taking into consideration the fact that PTH concentrations are not constant but increase during pregnancy, the present study aimed to evaluate PTH levels in two different stages of pregnancy (first and third trimesters) among women living in Switzerland. Besides the effect of circulating 25(OH)D concentrations on PTH levels, we aimed to investigate additional factors, such as socioeconomic determinants and lifestyle factors, that might modulate PTH concentrations.

## 2. Materials and Methods

### 2.1. Study Population

The study was conducted between August 2014 and June 2016 at the Clinic of Obstetrics of the University Hospital Zurich (USZ). Pregnant women were recruited in their first trimester of pregnancy while attending their first routine antenatal examination (pregnancy weeks 5–12) or in their third trimester of pregnancy during their last routine examination, which takes place within days before delivery (pregnancy weeks 37–42). Inclusion criteria were pregnancy, minimum age of 18 years, current residence in Switzerland for at least six months before the start of pregnancy, and fluency in German, French, Italian, or English. The exclusion criteria of the study participants were multiple pregnancies (e.g., twin or triplet), age below 18 years, known or suspected drug or alcohol abuse, HIV infection, or known conditions of a history of parathyroid, renal, or liver disease, chronic malabsorption syndromes, or granuloma-forming disorders during patient anamnesis, considering that they may alter PTH concentrations. In our study, none of the women were using diuretics or hormones influencing PTH metabolism. Progesterone for pregnancy support due to in vitro fertilization treatment was not an exclusion criterion. Our final sample consisted of 407 pregnant women.

This study was not representative of the whole Swiss population; however, we chose the USZ based on socio-demographic and cultural background heterogeneity that the city of Zurich offers, given the large ethnically diverse population (32.5% foreign resident population in 2018) [30]. This study was approved by the ethics committee of the canton of Zurich, Switzerland (KEK-ZH-Nr. 2013-0213), and all participants provided written informed consent before enrollment.

### 2.2. Measurements of Serum 25(OH)D and PTH

At enrollment, a 10 mL blood sample was drawn from each participant and analyzed within hours following withdrawal in the Institute of Clinical Chemistry at the USZ. The vitamin D status was evaluated by measuring the concentrations of 25(OH)D, the most informative biomarker of vitamin D status reflecting vitamin D obtained from both dietary intake and UV skin synthesis [31]. Total 25(OH)D was measured using the vitamin D total-analysis Roche Cobas^®^ electrochemiluminescence immunoassay (Roche Diagnostics, Basel, Switzerland). The method has a detection range of 7.5–175 nmol/L for 25(OH)D and a variation coefficient of 2.2–6.8%.

Vitamin D deficiency was defined as a 25(OH)D concentration of <20 ng/mL (50 nmol/L), while a serum 25(OH)D concentration of ≥20 ng/mL (50 nmol/L) was considered sufficient in line with the recommendations of the Institute of Medicine (IOM) [32]. Since there is no universally accepted definition of vitamin D deficiency, we decided to adopt this frequently used cut point. The serum concentrations of PTH (ng/L) were determined using chemoluminescence immunoassay (Roche Diagnostics, Basel, Switzerland), with coefficients of variations of 2.2% (41.1 ng/L) and 2.3% (146.5 ng/L). This assay measures intact PTH (PTH 1-84) but cross-reacts with PTH 7-84, and hence measures both.

### 2.3. Covariates

A questionnaire and the data collected by the physicians at enrollment were used to obtain information regarding potential determinants of PTH levels in the current analysis. The variables listed below were retrieved: maternal age (years), week of pregnancy, nulliparity (yes vs. no), first pregnancy (yes vs. no), self-reported body mass index BMI (kg/m^2^) before pregnancy, body weight gained during pregnancy (kg), measured BMI near term (kg/m^2^), and educational level of the participant and her partner, classified according the International Standard Classification of Education [33]; levels were divided into the following categories: less than compulsory education vs. low education (compulsory education) vs. middle education (secondary education) vs. high education (tertiary education). Furthermore, information about the country of origin categorized based on the World Bank Map (namely, group 1: Switzerland and Germany; group 2: North America and Europe, Central Asia, and New Zealand; group 3: South Europe, Australia, and Latin America; group 4: South and East Asia and Pacific; and group 5: Africa and the Middle East), number of days per week spent at least 1 h outdoors in the past 6 months, and smoking status (never vs. former vs. current) were requested. Other variables included the season of blood collection (winter (21 December–20 March) vs. spring (21 March–20 June) vs. summer (21 June–20 September) vs. autumn (21 September–20 December)), frequency of sunscreen use when exposed to the sun in summer (never vs. sometimes vs. always), consumption of fish (salmon, herring, sardine, mackerel, or tuna) at least once a week (yes vs. no), intake of vitamin-D-containing supplements (yes vs. no), iron supplements (yes vs. no) and intake of multivitamin supplementation (yes vs. no), and skin color (light vs. dark). Skin color was assessed using a five-level scale classification by Fitzpatrick [34]. Briefly, participants were shown a picture of different skin color types (I-V) and had to rate their type. The same evaluation was done simultaneously by the physician. In a second step, participants were asked to describe how their untanned skin reacts to sun exposure (if exposed in the early summer at noon for 45–60 min). Based on their skin color type and skin-tanning evaluations, women estimated their skin phototypes. If the classification of the participant and the physician disagreed, a rounded arithmetic mean was taken. To account for the small number of women in groups IV and V, the skin color variable was dichotomized into light skin color (Fitzpatrick levels I to III) and dark skin color (Fitzpatrick levels IV and V). In addition, skin type was also determined by calculating the arithmetic mean of the melanin index (measured three times) during the interview and then was categorized in quartiles.

### 2.4. Statistical Analyses

All statistical analyses graphs were conducted using STATA software version 16 (College Station, Texas). Continuous variables were described as the mean with standard deviation (if normally distributed) or the median with an interquartile range (if skewed), while categorical variables were given as frequencies with percentages. The Mann–Whitney U or the Kruskal–Wallis test was used to compare PTH levels between comparison groups. Logarithmic transformation (log_10_) of PTH and 25(OH)D levels was performed because of skewed distribution. To examine the association between serum concentrations of 25(OH)D and PTH, the Spearman correlation coefficient was applied, and to investigate whether there was an inflection point in the 25(OH)D–PTH association curve, we used locally weighted estimated scatterplot smoothing (LOWESS) plots [35]. Hockey-stick regression [36] with PTH (ng/mL) as the dependent variable was used to identify a change point in 25(OH)D, where PTH reached a plateau and was maximally suppressed. The Chow test was used to test for a structural break in the association curve at the identified change point. Multiple imputations with chained equations (*m* = 25) were performed to deal with missing values, assuming data were missing at random. Twenty-five imputed data sets were created based on complete data variables. We examined univariate associations of PTH with potential determinants in the first and third trimesters of pregnancy using simple linear regressions. Several determinants have been suggested by prior research in the general population, such as the BMI, ethnicity, smoking, season, and age, to be associated with PTH concentrations [2,3,37]. The BMI has been reported to be inversely associated with vitamin D status and might affect PTH levels mediated by its effects on vitamin D [38,39]. The BMI and obesity are inversely related to the socioeconomic status; furthermore, evidence suggests an association between a lower socioeconomic status and a higher risk of diet-related conditions [40]. Thus, socioeconomic status might affect PTH through diet and be indirectly mediated by vitamin D. In our analysis, we used the education of the partner as a proxy woman’s socio-economic position. A lower educational level is also associated with poor diet quality and vitamin D deficiency [41,42], which could affect PTH levels through vitamin D but also diet. The following list of variables was included in the simple regression analysis: age, week of pregnancy, nulliparity, BMI before pregnancy, BMI near term, skin color, country of origin, education level achieved by the mother, education level achieved by the partner, smoking status, season, days spent in the sun, use of sunscreen, fish consumption and intake of vitamin-D-containing supplements, iron supplements, and multivitamin intake. In addition, we performed multiple linear regression using all significant potential determinants adding clinically relevant variables for PTH except variables showing high collinearity. Collinearity was defined as a Pearson’s correlation coefficient above 0.6 between continuous variables and a Cramér’s V above 0.6 between categorical variables. Correlation coefficients and Cramér’s V were calculated on complete cases. The selection of the final models for multivariable adjustment for each trimester was performed using the Akaike information criteria (AIC) score that was calculated on complete cases. Multiple linear regression analysis generated beta coefficients (β) and 95% confidence intervals (CIs). A *p*-value of <0.05 was considered statistically significant.

## 3. Results

### 3.1. General Characteristics of the Studied Population

A total of 407 women were included in the study, of which 204 were in the first and 203 women in the third trimester of pregnancy. The characteristics and lifestyle factors of the women are summarized in Table 1. Overall, the mean age at blood draw was 30.2 and 33.6 years in early and late pregnancy, respectively; 21.6% of women in early pregnancy and 33.3% of women in late pregnancy were of German or Swiss origin; and the vast majority of women in both groups had secondary or tertiary education (83.3% and 80.1%, respectively). Among the participants in the first trimester of pregnancy (week of pregnancy, median = 8 weeks), 74% were classified as light skin colored and spent a median of 2 d/week regularly in the sun, during which at least 1 h/day was spent in the sun. Of women in the third trimester of pregnancy (week of pregnancy, median = 38 weeks), 85.6% were classified as light skin colored and regularly spent time in the sun (median of 4 d/week, during which at least 1 h was spent in the sun). In early and late pregnancy, median serum 25(OH)D concentrations were below the 20 ng/mL (50 nmol/L) cutoff for vitamin D deficiency, 17.1 ng/mL (42.8 nmol/L) and 17.9 ng/mL (44.8 nmol/L), respectively. In addition, 63% of the women in the first trimester and 54% of women in the third trimester of pregnancy in our study cohort had lower serum 25(OH)D concentrations than the indicated diagnostic criteria for vitamin D sufficiency.

### 3.2. PTH Concentrations by Pregnancy Trimester, Country of Origin, Serum Vitamin D Levels, and Season

**Figure 1:**
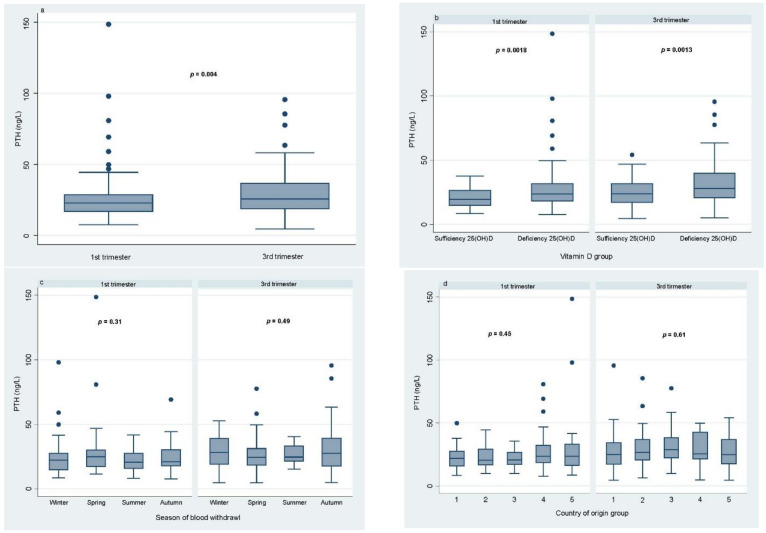
Serum PTH concentrations (ng/L) by (**a**) 1st and 3rd trimesters of pregnancy, (**b**) vitamin D group (sufficient = 25(OH)D ≥ 20 ng/mL (50 nmol/L) or deficient = 25(OH)D < 20 ng/mL), (**c**) season of blood withdrawal, and (**d**) country of origin. Boxplots represent the median, 1st, and 3rd quartiles of the complete cases; bars indicate minimum and maximum; dots indicate outliers.

**Figure 2:**
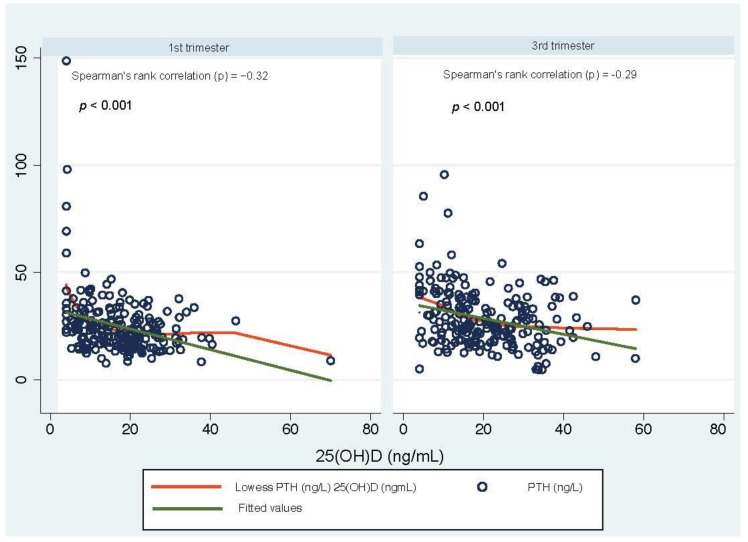
Scatterplot of PTH (ng/L) versus 25(OH)D (ng/mL) concentrations with smoothing fit. Left: 1st trimester group: Right: 3rd trimester group.

Serum PTH levels in pregnant women by (a) pregnancy trimester, (b) category of vitamin D concentrations, (c) season of blood draw, and (d) country of origin and are shown in Figure 1. Median PTH serum concentrations were statistically lower in early pregnancy (22.75 ng/L) compared to late pregnancy (25.7 ng/L) (*p* = 0.004; Figure 1a). Women with vitamin D deficiency had significantly higher PTH concentrations compared to women with sufficient levels of vitamin D in both groups (*p* = 0.002 in early pregnancy and *p* = 0.001 in late pregnancy; Figure 1b). No statistically significant seasonal differences in PTH concentrations were observed in early or late pregnancy (*p* = 0.3 and *p* = 0.5, respectively; Figure 1c). There was no statistically significant difference between median PTH concentrations for the five groups of country of origin in early and late pregnancy (*p* = 0.4 and *p* = 0.6, respectively; Figure 1d). The relationship between PTH and 25(OH)D is depicted in Figure 2. A negative correlation between PTH and 25(OH)D was observed for both the first and the third trimester (Spearman’s correlation *ρ* = −0.32 and *ρ* = −0.29, *p* < 0.0001; Figure 2). For the first trimester of pregnancy, the smoothing fit did not suggest a potential cutoff point, indicating a change in the direction of the association between PTH and 25(OH)D. The hockey-stick regression and Chow test used to identify such threshold confirmed that there was no changing point (*p* = 0.21, see Supplementary Material Table S1). On the other hand, in the third trimester of pregnancy, the smoothing fit suggested a potential cutoff point, indicating a change in the direction of the association between PTH and 25(OH)D between 18 and 20 ng/mL. When applying the hockey-stick regression and Chow test to identify the cutoff value, we determined that 18.9 ng/mL (47 nmol/L) was the cutoff concentration of 25(OH)D at which the difference in slopes of the regression lines below and above this point reached the maximum value and above which PTH concentrations reached a plateau (i.e., slope did not differ from 0). Below and above 25(OH)D concentrations of 18.9  ng/mL, the slopes were −1.128 (different from 0; *p* = 0.001) and −0.18 (*p* = 0.423), respectively. The difference between the slopes of the two regression lines was −0.948 (*p* = 0.005; see Supplementary Material Table S2).

### 3.3. Determinants of PTH Concentrations: Multiple Regression Results

Determinants of PTH concentrations in the first and third trimesters of pregnancy were evaluated by linear regression using different adjustment models, of which the best-fitting models (according to AIC) are summarized in Table 2 for early pregnancy and in Table 3 for late pregnancy.

After testing for collinearity between all variables, the following pairs of variables were considered correlated: gravidity and nulliparity (Cramér’s V = 0.81), education of the mother and education of the partner (Cramér’s V = 0.78), skin color and country of origin (Cramér’s V = 0.64), and finally BMI before pregnancy and BMI at enrolment (Pearson’s correlation coefficient  =  0.98). After comparing several models and AIC scores to identify the best compromise between statistical validity and clinical relevance of the determinants, we decided to exclude BMI before pregnancy, education of the mother, skin color, and gravidity, as we considered them the least informative. Our decision to exclude from the analyses the variables education of the mother and skin color was also supported by the lack of association with PTH in the univariate analysis, while we decided to exclude BMI before pregnancy and gravidity based only on the AIC score, indicating the best model. Multiple regression analysis showed that statistically significant determinants for PTH levels were 25(OH)D concentrations, education of the partner, nulliparity, outdoor activity evaluated as days per week spent at least 1 h outdoor in the past half year, and season of blood draw for women in early pregnancy. In late pregnancy, vitamin D concentrations and country of origin were the only determinants found to be statistically significant (Table 3).

According to these models, a 1% increase in 25(OH)D levels is estimated to result in about 0.29% and 0.27% lower PTH levels in the first and third trimester groups, respectively. Women’s country of origin was a statistically significant determinant in late pregnancy but not in early pregnancy. Specifically, women from African and Middle Eastern origin had 24.6% lower PTH concentrations. Middle or high education of the partner resulted in statistically lower PTH levels compared to less than compulsory education of partners for women in late pregnancy (24.6% and 24.6%, respectively). In early pregnancy, PTH was 17% higher in women who had given birth before compared to nulliparous participants. Outdoor activity resulted in lower PTH concentrations (Table 2). Blood draw in spring resulted in statistically significant higher PTH levels (17%) compared to winter blood draw for women in early pregnancy (Table 2) In addition, we examined age, BMI, body weight gain, and iron supplement and multivitamin intake and found no significant associations between these variables and the PTH level in early or late pregnancy.

## 4. Discussion

This study is one of few investigating PTH levels among pregnant women in the early and late stages of gestation. To the best of our knowledge, PTH levels and the determinants associated with this hormone during pregnancy have not been investigated in the Swiss population. We observed a negative correlation between PTH and vitamin D concentrations, which is in agreement with findings of previous studies [3,22,23,43,44,45]. The effect of vitamin D on the PTH concentration in the third trimester was more strongly perceptible when the 25(OH)D concentration was <18.9 ng/mL (47.3 nmol/L). Above that concentration, it appears that other determinants have a stronger effect on the PTH concentration. The difference in the slopes of the lines of regression above and below this point (*p* = 0.005) is statistically significant. Furthermore, the slope of the regression line between plasma PTH and 25(OH)D concentrations below 18.9 ng/mL (47.3 nmol/L) is significantly different from zero (*p =* 0.001), whereas above these concentrations, the slope of the regression line does not differ from zero (*p* = 0.423). In addition, our estimate is similar to estimates obtained in populations in other studies performed during pregnancy and the postpartum period and in agreement with the IOM’s standard definition of vitamin D deficiency in adults of 25(OH)D concentrations ≤20 ng/mL (50 nmol/L) [23,46]. Of note, 63% of the women in the first trimester and 54% of women in the third trimester of pregnancy in our study cohort had lower serum 25(OH)D levels than the indicated diagnostic criteria for vitamin D sufficiency that the Endocrine Society has endorsed in non-pregnant adults [47]. This emphasizes the importance of appropriate counseling and vitamin D supplementation in pregnancy, as previously concluded in studies conducted in the same cohort [48,49]. Given sample restrictions, we could not stratify our analysis in the vitamin-D-sufficient or the vitamin-D-deficient group to look more in depth at how that could affect our study results regarding the predictors of PTH concentrations. Several determinants of PTH concentrations in the general population and during pregnancy, such as season [22], ethnicity [50], age [20,51], and gestational age [26,44], have previously been reported. Haddow et al. reported higher PTH concentrations in African American women compared to Caucasian women [22]. In contrast, our results showed that women of African and Middle Eastern origin have lower PTH levels than Swiss and German women in late pregnancy, despite lower 25(OH)D concentrations, as shown previously [48,49,52]. Previous reports have suggested a lower threshold for PTH secretion in response to vitamin D status in black compared to white women [53,54]. While studies are more consistent reporting a 25(OH)D threshold for white women close to 50 nmol/L, they suggest a threshold between 37 to 40 nmol/L for dark-skinned women [54]. Furthermore, substantive ethnic differences have been reported in the calcium economy. Blacks appear to have superior renal reabsorption of calcium, have relative skeletal resistance to PTH, and require less calcium intake per day than whites [55]. We can only speculate at this point that our unexpected results of higher PTH concentrations in African American women could be related to these racial differences in the PTH and 25(OH)D threshold and calcium economy. Unfortunately, we could examine racial differences of this threshold due to small numbers of African American women in our sample.

It is generally accepted that elevated PTH concentrations are an indicator of vitamin D deficiency; nevertheless, there are other factors that are involved in the physiological changes in PTH. Herein, we investigated other potential factors determining PTH concentrations using multiple regression models. These models indicated that 25(OH)D concentrations are a statistically significant determinant of PTH levels in both early and late pregnancy. Education of the partner, multiparity, season, and outdoor activity were also statistically significant in early pregnancy, whereas country of origin was a statistically significant determinant of PTH in late pregnancy. Studies in the general population and in pregnant women have demonstrated an association between education and vitamin D levels, such that better education is associated with higher levels of vitamin D [42,56,57]. In our analysis, a higher education level of partners resulted in significantly lower PTH levels. Education of the partner might in some cases be a better indicator of a woman’s socio-economic position than her own education and, thus, influence her lifestyle and health behavior [58]. In our analysis, having given birth previously was associated with significantly higher PTH levels. The reasons for this finding are unclear, and further research is needed to elucidate the mechanism whereby multiparity of women might affect PTH levels. We observed that a higher median number of days per week spent with at least 1 h outdoors in the past 6 months was also associated with higher PTH levels. From a previous report on the same group of women, it was observed that women that were vitamin D deficient spent more time outside compared to women with sufficient vitamin D levels [52]. This probably represents a behavioral change during pregnancy compared to before because of their vitamin D deficiency and could explain the observed higher PTH concentrations in these women. Against our expectations, PTH levels were significantly higher in spring compared to winter but not in summer or autumn. The observed higher PTH concentrations in spring compared to winter could be explained by the fact that the lowest concentration of vitamin D is, indeed, observed in spring due to the longer period of lacking sun exposure. Following the same reasoning, we would have expected, a priori, lower significant PTH concentrations in summer and autumn, but our results were not significant. A plausible explanation could be that other plasma factors, such as phosphate or calcium, that were not accounted for could impact PTH levels during pregnancy independent from serum 25(OH)D levels. Phosphate might effect PTH; depending on the extracellular phosphate balance, it might indirectly suppress or directly increase PTH, as has recently been reported [59,60]. Plasma calcium levels are inversely associated with PTH [59,61]. Furthermore, previous research has suggested a biphasic pattern of PTH, showing a late-afternoon/early-evening rise and fall and a broader, longer-lasting increase late evening/early morning, reaching the lowest levels mid-morning [62]. We were unable to account for the circadian rhythm of PTH and how it could impact PTH levels, since blood samples were taken during routine visits at the clinic and, hence, at different times of the day. PTH is affected by maternal dietary calcium intake as well as PTHrP [9]. Previous longitudinal studies suggest that PTHrP begins to rise from the third to the thirteenth week of gestation and increases threefold per term in comparison to the baseline pre-pregnancy level [63,64]. The significant rise in PTHrP may upregulate calcitriol and suppress PTH [9]. The effect of calcium intake and 25(OH)D levels on PTH levels has been previously reported [18]. Steingrimsdottir et al. observed that serum PTH varied by calcium intake at low serum 25(OH)D levels (<10 ng/mL). Calcium intake of less than 800 mg/d vs. more than 1200 mg/d was significantly associated with higher serum PTH [18]. Furthermore, calcium intake varies by season [65,66]. Since dietary assessment of calcium intake (with or without supplementation) and measurement of PTHrP were not included in our study, we could not examine these relationships with PTH levels.

There are several limitations to our study that should be acknowledged: low generalizability considering the small sample size, the specific population of just one hospital, and self-selection of study participants. However, more than two-thirds of the women approached by the recruiting physicians participated in this study. The cross-sectional study design is another limitation that does not allow for inferences of causality. Our models accounted for 33% (first trimester) and 28% (third trimester) of the variability in PTH concentrations in our study population. Thus, there are likely other factors that could influence PTH levels, such as serum 1.25(OH)D [1], PTHrP, calcium intake [9], and plasma phosphate or calcium, as suggested by the previous literature, that we were unable to examine in our study. The PTH assay measures intact PTH (PTH 1-84) but cross-reacts with PTH 7-84. Hence, small differences in PTH concentrations among women with concentrations in the normal range may not have reflected differences in the active hormone.

## 5. Conclusions

The determinants associated with variations in PTH concentrations in the early and late stages of pregnancy in women recruited at the University Hospital Zurich were investigated using multiple regression analysis. Our analyses support a negative correlation between PTH and vitamin D concentrations during pregnancy and suggest that in the third trimester of pregnancy, a 25(OH)D level of 18.9 ng/mL could be the criterion for 25(OH)D deficiency that serves as an inflection point for the maximal suppression of PTH. Our multiple regression models confirmed that 25(OH)D concentrations were a statistically significant determinant of PTH levels in both early and late pregnancy. Additionally, season, outdoor activity, multiparity, and education of the partner were significant determinants of PTH concentrations in early pregnancy, whereas the country of origin was a predictor in late pregnancy in this group of women living in Switzerland. Besides vitamin D, factors that were associated with PTH concentrations in our study were non-modifiable (multiparity, season, education of the partner). These factors and their effect on PTH appear to be vastly determined by 25(OH)D; however, we cannot exclude that they might also affect PTH through other mechanisms besides 25(OH)D.

## Figures and Tables

**Table 1:** General characteristics of pregnant women in first and third trimesters of pregnancy.

	1st Trimester		3rd Trimester		Missing *n* (%)
*n* (%)	204	50.12	203	49.88	
PTH ng/L, median (Q1, Q3)	22.75	16.9, 28.6	25.7	18.8, 36.6	
25(OH)D ng/mL, median (Q1, Q3)	17.1	9.75, 22.3	17.9	11.9, 28.1	
25(OH)D nmol/L, median (Q1, Q3)	42.8	24.4, 55.8	44.8	29.8, 70.3	
Vitamin D deficiency, *n* (%)	129	63.24	111	54.68	
Skin color, *n* (%) *					1 (0.25)
Light	151	74.02	173 |	85.64	
Dark	53	25.98	29	14.36	
Melanin levels, median (Q1, Q3)	36.45	30.2–39.58	32.94	29.35–34.67	2 (0.49)
Age, mean (SD)	30.20	4.85	33.59	5.06	
Week of pregnancy, median (Q1, Q3)	8	7.59	38	38.38	
Parity, *n* (%) nulliparous	108	52.94	60	29.56	
Gravidity, *n* (%) first pregnancy	84	41.18	51	25.12	
BMI (kg/m^2^) before pregnancy, median (Q1, Q3)	22.90	19.94, 24.87	24.30	21.23, 25.71	23 (5.65)
BMI (kg/m^2^) current, median (Q1, Q3)	23.17	20.42, 25.74	29.00	25.59, 30.85	6 (1.47)
Country of origin, *n* (%)					2 (0.49)
Switzerland and Germany	44	21.57	67	33.3	
North America and Europe, Caucasus, Central Asia, and New Zealand (without Switzerland and Germany)	63	30.88	62	30.85	
South Europe, Australia, Latin America, and the Caribbean	30	14.71	30	14.93	
South and East Asia, Pacific	32	15.69	18	8.96	
Africa, and the Middle East	35	17.16	24	11.94	
Educational level achieved, *n* (%)					1 (0.25)
Less than compulsory education	13	6.37	9	4.46	
Low education	21	10.29	31	15.35	
Middle education	67	32.84	64	31.68	
High education	103	50.49	98	48.51	
Educational level achieved of the partner, *n* (%)					11 (2.07)
Less than compulsory education	14	6.93	11	5.67	
Low education	21	10.4	19	9.79	
Middle education	77	38.12	71	36.6	
High education	90	44.55	93	47.94	
Smoking status, *n* (%)					3 (0.74)
Never smoker	121	59.31	122	60.4	
Ever smoker	62	30.39	61	30.2	
Current smoker	21	10.3	19	9.41	
Season, *n* (%)					3 (0.74)
Winter	39	19.1	64	32	
Spring	46	22.55	78	39	
Summer	50	24.5	18	9	
Fall	69	33.82	40	20	
Days per week spent at least 1 h outdoor (Q1, Q3)	2	2, 7	4	2, 7	8 (1.97)
Using sun protection in summer, *n* (%)					2 (0.49)
Never	50	24.63	42	20.79	
Sometimes	75	38.42	81	40.1	
Always	78	36.95	79	39.11	
Fish consumption at least once per week, %	96	47.52	109	54.23	6 (1.47)
Vitamin D supplement intake, *n* (%)	17	8.33	32	15.76	
Multivitamin supplement intake, *n* (%)	86	42.16	127	62.56	
Iron supplement intake, *n* (%)	28	14.07	96	60	48 (11.8)

BMI, body mass index; PTH, parathyroid hormone; 25(OH)D, 25-hydroxyvitaminD; Q1, Q3, first, third quartile. * Light skin color defined as values I–III from the Fitzpatrick scale. Dark skin color defined as value IV-V from the Fitz Patrick scale.

**Table 2:** Multiple regression analysis and determinants for log PTH concentrations in the 1st trimester.

Factors	Coef. *	95% CI	
Log 25(OH)D ^l^	−0.289	−0.401	−0.178
Age	0.010	−0.002	0.022
Country of origin			
Switzerland and Germany	ref		
North America and Europe, Caucasus, Central Asia, and New Zealand (without Switzerland and Germany)	0.018	−0.128	0.165
South Europe, Australia, Latin America, and the Caribbean	−0.030	−0.205	0.145
South and East Asia and the Pacific	−0.047	−0.243	0.149
Africa and the Middle East	−0.176	−0.371	0.019
Education ^1^			
Less than compulsory education	ref		
Low education	−0.241	−0.506	0.024
Middle education	−0.405	−0.644	−0.166
High education	−0.378	−0.625	−0.131
Nulliparity			
Yes	ref		
No	0.166	0.057	0.274
Days per week spent at least 1 h outdoor	0.020	0.001	0.039
BMI	0.004	−0.008	0.017
Multivitamin supplementation	−0.081	−0.195	0.034
Season			
Winter	ref		
Spring	0.165	0.002	0.327
Summer	0.098	−0.061	0.257
Autumn	0.093	−0.054	0.239

BMI, body mass index; PTH, parathyroid hormone; 25(OH)D, 25-hydroxyvitaminD ^1^ Education levels of the partner. * Model selected using AIC-based forward backward selection, estimated on the complete data. AIC = 173.70 Number of study participants is 204. *R*^2^ = 0.33 for the regression model.

**Table 3:** Multiple regression analysis and determinants for log PTH concentrations in the 3rd trimester.

Factors	Coef. *	[95% CI]	
Log 25(OH)D	−0.277	−0.404	−0.149
Age	0.003	−0.012	0.017
Country of origin			
Switzerland and Germany	ref		
North America and Europe, Caucasus, Central Asia, and New Zealand (without Switzerland and Germany)	−0.022	−0.201	0.157
South Europe, Australia, Latin America, and the Caribbean	0.043	−0.178	0.264
South and East Asia and the Pacific	−0.103	−0.365	0.159
Africa and the Middle East	−0.246	−0.491	−0.01
Education ^1^			
Less than compulsory education	ref		
Low education	0.036	−0.353	0.425
Middle education	−0.103	−0.433	0.227
High education	−0.015	−0.352	0.322
Sun protection			
Never	ref		
Sometimes	−0.111	−0.314	0.092
Always	−0.116	−0.323	0.091
BMI	−0.002	−0.017	0.013
Season			
Winter	ref		
Spring	−0.056	−0.229	0.116
Summer	0.102	−0.169	0.372
Autumn	0.055	−0.146	0.256
Iron supplementation	0.095	−0.071	0.261

BMI, body mass index; PTH, parathyroid hormone; 25(OH)D, 25-hydroxyvitaminD ^1^ Education levels of the partner. * Model selected using AIC-based forward backward selection, estimated on the complete data. AIC = 184.47 Number of study participants is 203. *R*^2^ = 0.28 for the regression model.

## Data Availability

The data are not publically available, but will be shared upon reasonable request.

## Supplementary Materials

The following are available online at https://www.mdpi.com/2072-6643/13/2/360/s1, Table S1: Hockey-stick regression models with PTH (ng/mL) as the dependent variable and 25(OH)D as the independent variable adjusted for regression variables in the first trimester, Table S2: Hockey-stick regression models with PTH (ng/mL) as the dependent variable and 25(OH)D as the independent variable adjusted for regression variables in the third trimester.
