# Supplementary material for: Parathyroid Hormone in Pregnancy: Vitamin D and Other Determinants
Source: Nutrients. 2021 Jan 25;13(2):360. doi: 10.3390/nu13020360 (PMC7911996; doi:10.3390/nu13020360)
Supplement: Supplementary file 1 [file nutrients-13-00360-s001.pdf]

Additional table 1. Hockey-stick regression models with PTH (ng/ml) as dependent variable and *25(OH)D as the independent variable adjusted for the regression variables* in the *first trimester*

	25(OH)D	[95% Conf	Interval]	P value
Constant	157.646*	16.196	299.095	0.029
Change-point of 25(OH)D	5.123*	3.613	6.634	0
Left slope	-24.909	-59.248	9.429	0.154
Right slope	-.143	-.374	.088	0.223
Country of origin	-.862	-2.329	.605	0.248
Education	-3.246	-5.369	-1.124	0.003
Nulliparity	3.477	.011	6.942	0.049
Outdoor activity	.506	-.127	1.139	0.117
BMI	.007	-.413	.426	0.974
Multivitamin supplements	-2.227	-5.983	1.529	0.244
Season	.383	-1.141	1.907	0.621
R^2	0.361			
Change-point p-value†	0.21			

*Definition of abbreviations: PTH= parathyroid hormone; R^2 = coefficient of determination. Table showing hockey-stick regression models with PTH as dependent variable. The variable **25(OH)D** with an added change-point is given in column heading. Left slope = coefficient for variable up to change-point. Right slope = coefficient for variable after change-point. *P-value<0.05. †P-value for structural change at identified change-point (Chow test).*

Additional table 2. Hockey-stick regression models with PTH (ng/ml) as dependent variable and *25(OH)D as the independent variable adjusted for the regression variables* in the *third trimester*

	25(OH)D	[95% Conf	Interval]	P value
**Constant**	37.117*	12.257	61.977	0.611
**Change-point of *25(OH)D***	18.924*	10.316	27.531	0
**Left slope**	-1.128*	-1.812	-.444	0.001
**Right slope**	-.18	-.622	.263	0.423
**Age**	.472	.019	.925	0.041
**Country of origin**	-1.111	-2.826	.603	0.202
**Education of the partner**	-1.549	-4.416	1.318	0.287
**Sun protection**	-2.451	-5.747	.846	0.144
**BMI**	.065	-.429	.558	0.379
**Season**	1.178	-.847	3.203	0.795
**Iron supplementation**	-1.159	-5.659	3.341	0.252
**R^2**	0.25			
**Change-point p-value†**	0.005*			

*Definition of abbreviations: PTH= parathyroid hormone; R^2 = coefficient of determination. Table showing hockey-stick regression models with PTH as dependent variable. The variable **25(OH)D** with an added change-point is given in column heading. Left slope = coefficient for variable up to change-point. Right slope = coefficient for variable after change-point. *P-value<0.05. †P-value for structural change at identified change-point (Chow test).*
